# Sources, seasonal cycling, and fate of plutonium in a seasonally stratified and radiologically contaminated pond

**DOI:** 10.1038/s41598-023-37276-w

**Published:** 2023-07-08

**Authors:** Naomi L. Wasserman, Nancy Merino, Fanny Coutelot, Daniel I. Kaplan, Brian A. Powell, Annie B. Kersting, Mavrik Zavarin

**Affiliations:** 1 Glenn T. Seaborg Institute, Physical and Life Sciences Directorate, Lawrence Livermore National Laboratory, 7000 East Ave, L-231, Livermore, CA 94550 USA; 2 Department of Environmental Engineering and Earth Sciences, Clemson University, Anderson, SC 29625 USA; 3 Center for Nuclear Environmental Engineering Sciences and Radioactive Waste Management, Clemson University, Anderson, SC 29625 USA; 4 Savannah River National Laboratory, Aiken, SC 29625 USA

**Keywords:** Element cycles, Limnology

## Abstract

Unlike short-term laboratory experiments, studies at sites historically contaminated with radionuclides can provide insight into contaminant migration behavior at environmentally-relevant decadal timescales. One such site is Pond B, a seasonally stratified reservoir within Savannah River Site (SC, USA) has low levels (μBq L^−1^) of plutonium in the water column. Here, we evaluate the origin of plutonium using high-precision isotope measurements, investigate the impact of water column geochemistry on plutonium cycling during different stratification periods, and re-evaluate long-term mass balance of plutonium in the pond. New isotopic data confirm that reactor-derived plutonium overwhelms input from Northern Hemisphere fallout at this site. Two suggested mechanisms for observed plutonium cycling in the water column include: (1) reductive dissolution of sediment-derived Fe(III)-(oxyhydr)oxides during seasonal stratification and (2) plutonium stabilization complexed strongly to Fe(III)-particulate organic matter (POM) complexes. While plutonium may be mobilized to a limited extent by stratification and reductive dissolution, peak plutonium concentrations are in shallow waters and associated with Fe(III)-POM at the inception of stratification. This suggests that plutonium release from sediments during stratification is not the dominant mechanism driving plutonium cycling in the pond. Importantly, our analysis suggests that the majority is retained in shallow sediments and may become increasingly recalcitrant.

## Introduction

Environmental releases of long-lived anthropogenic radionuclides, especially abundant in the early decades of the atomic age, pose a persistent health and environmental risk world-wide^1^. As we approach the 80th anniversary of the first nuclear test, the fate of radioactive isotopes in the environment on decade-to-century timescales becomes increasingly important. Monitoring efforts at numerous sites have revealed complex influences from hydrological, geochemical, and microbial processes controlling radionuclide mobility^2–4^. Few other radionuclides exhibit this complexity more than plutonium, which displays oxidation state-dependent solubility^5,6^, can complex strongly to organic matter^7^, and can form intrinsic colloids and pseudo-colloids (e.g. iron-bearing and clay minerals)^8–12^. More common isotopes of plutonium (^238^Pu [t_1/2_ = 87.7 y], ^239^Pu [t_1/2_ = 24,100 y], and ^240^Pu [t_1/2_ = 6,561 y]) have long half-lives, and therefore, combined with their highly toxic nature pose long-term health risks.

This study focuses on the seasonal and long-term biogeochemical processes that directly and indirectly affect trace plutonium mobility in the water column of the monomictic pond, Pond B. Beginning in 1961, the pond functioned as a cooling reservoir for thermal discharge from Savannah River Site’s R Reactor^13^. Documented releases from R Reactor before and during utilization of Pond B are thought to have contributed to the persistent low levels (μBq L^−1^) of plutonium within the Pond B water column since the reactor’s closure in 1964^13,14^.

While the sole origin of plutonium from R Reactor has been implied in previous studies, no facility documents or research has excluded other inputs. From 1954 to 1989, hundreds of GBq of plutonium were released atmospherically and in local surface waters from various facilities at Savannah River Site^15^. Therefore, external sources of plutonium outside of R Reactor releases cannot be ruled out. We utilize high precision plutonium isotope ratio measurements of water column and upstream samples to constrain the origin of plutonium in Pond B.

Plutonium cycling within Pond B displays distinct seasonality due to summer stratification and subsequent holomixis in the late fall. Previous studies of water column plutonium concentrations concluded that summer anoxia drove plutonium release from reductive dissolution of Fe(III)-(oxyhydr)oxides^13,16^. However, others have argued that plutonium cycling is associated with organic matter complexation and is dominantly remobilized in late winter or early spring^14^. In this study, we examine the impact of these two mechanisms of seasonal remobilization on the plutonium water column budget. In addition, we draw on historical data to assess long-term plutonium fluxes within the pond system. As such, this field site presents a unique opportunity to examine the seasonal controls on remobilization and long-term ecological cycling of plutonium in natural systems. This study represents the first of a two-part study. In part II, we focus specifically on microbial community dynamics and impacts on plutonium and iron cycling^17^.

### Site description

**Figure 1:**
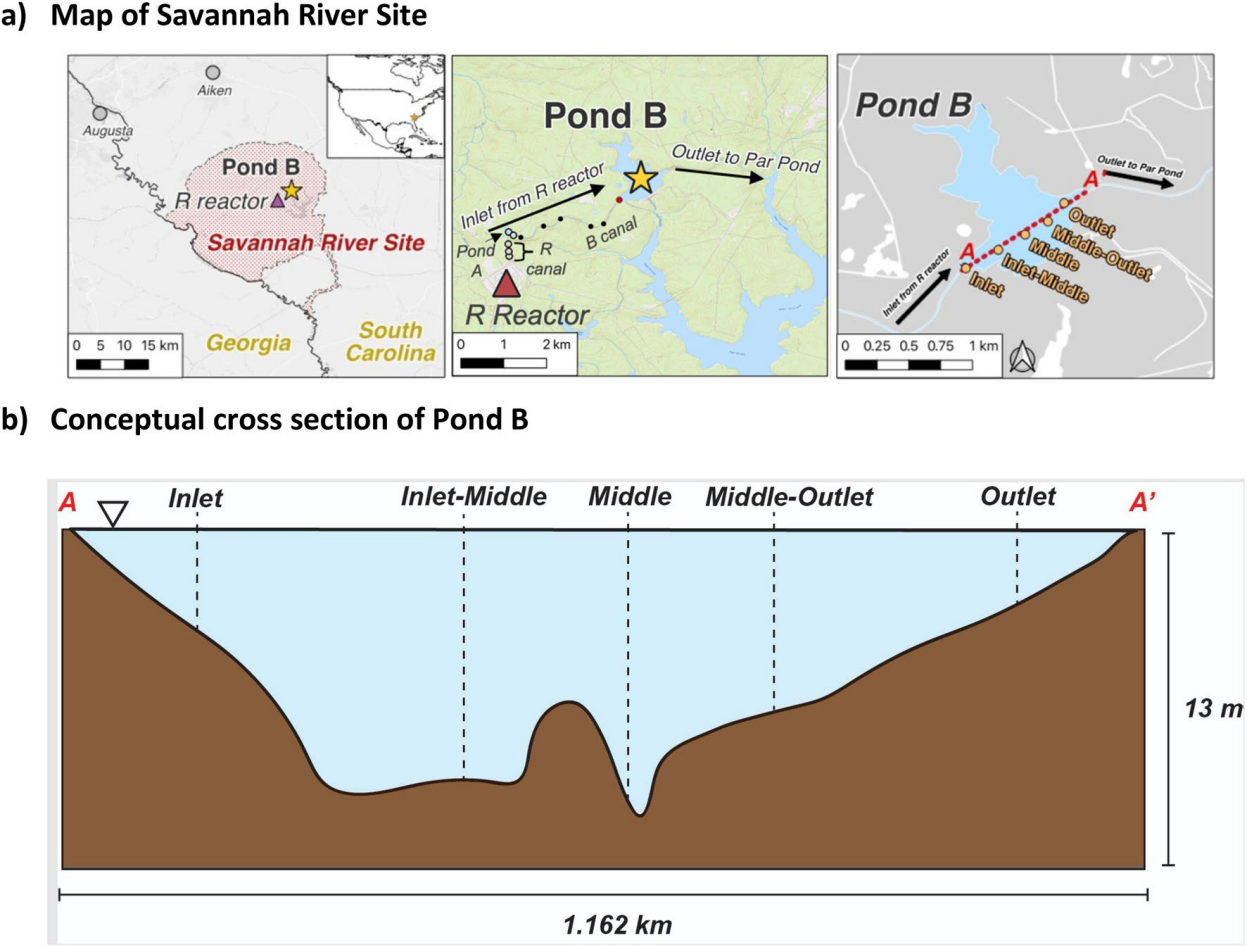
(**a**) Map of Savannah River Site (left), Pond B and R Reactor (center), and Pond B (right). R Reactor (red triangle) is located upstream of Pond B (yellow star). Empty circles denote sediment sampling locations along R canal and Pond A, while black circles indicate sediments sampled from B canal. Pond B water column sampling locations (yellow circles) lie along a transect (A–A′) from the inlet of Pond B to the outlet. (**b**) Conceptual cross section of sampling locations along transect A–A′ in Pond B. The Middle location represents the deepest extent of our sampling (~ 10 m).

Pond B is a dammed tributary, constructed in 1961 to serve as a reservoir within the secondary cooling loop for R Reactor, which produced weapons-grade plutonium and tritium until 1964 (Fig. 1a). During active pumping, it is estimated that water flowed from the reactor to Pond B at 11 m^3^ s^−1^^18^. In 1957, prior to the incorporation of Pond B into the secondary cooling loop, 1.11 × 10^10^ Bq of ^239+240^Pu and ^238^Pu were released into a seepage basin north of R Reactor^19^. This seepage basin was later converted to R Canal (Fig. 1a), which may have provided a source of plutonium to downstream bodies of water, like Pond B^20^. It is estimated that the ^239+240^Pu inventory released into Pond B was 4.3 × 10^8^ Bq^21^. Additional documented direct releases of radionuclides from fuel element and primary cooling system leakages as liquid effluent in R Reactor occurred in 1963 and 1964. Inventories of these releases include 1.9 × 10^15^ Bq ^3^H, 5.7 × 10^12^ Bq ^137^Cs, and 4.4 × 10^11^ Bq ^90^Sr, among other isotopes^15^. No plutonium was documented as part of this inventory, however, given the operations of the plant at the time and the source of the spill, it is likely that plutonium isotopes were also released. After reactor operations ceased in 1964, high pumping rates through Pond B ended and the pond has since remained relatively unperturbed hydrologically. A more extensive contaminant history is reviewed in Coutelot et al. (2022)^4^.

Pond B (0.87 km^2^) maintains a maximum depth of 12 m (Fig. 1b). Observations at the Pond for the past 4 years indicate that precipitation, overland flow from the surrounding watershed, and storm induced flow from R canal are the primary water inputs to the pond. Water exits the pond through a small stream with outflowing water on the east side of the pond and through releases from the toe of the dam. Sediments underlying the pond, reflective of the Carolina Sandhills physiographic region, are predominantly sandy with areas of higher accumulation of organic matter and kaolinitic clays^4,22^. As a monomictic pond, Pond B begins to stratify thermally in March and reaches maximum stratification by June. As the surface waters cool sufficiently and become denser, the pond overturns in late fall resulting in the water column remaining relatively oxic and thermally homogenous throughout the winter. During stratification, shallow waters have lower conductivities relative to the anoxic water layer^14^. On average, the waters are slightly acidic (pH 5 to 7)^23^.

## Methods

### Field sampling

From 2019 to 2021, in-situ water column measurements were collected at the Inlet, Inlet-Middle, Middle, Middle-Outlet and Outlet locations (Fig. 1b**, **Supplementary Fig. 1). Two multiparameter sondes (Aqua TROLL 600, In-situ) were connected in-tandem and equipped with the following probes/sensors: temperature/conductivity, pH/Oxidation–reduction potential (ORP), turbidity, dissolved oxygen (RDO sensor), and chlorophyll A. Water column measurements were taken in the following months (year): February (2021), March (2020), June (2019), October (2019), and December (2019). Most but not all locations were sampled during each of the campaigns.

Depth-discrete sampling was also performed by pumping water to the surface at 1 m intervals via tubing (Masterflex C-Flex® ULTRA) connected to the sonde. At each depth interval, water was pumped for about 5 min before filtering through 0.22 µm Sterivex™ filters (MilliporeSigma) until clogged (about 50–100 mL). The filtrate was retained for analysis of total organic carbon (TOC; stored in amber TraceClean® Type I borosilicate glass vials) and major anions (stored in trace-metal-free 50 mL Falcon tubes), and samples were transported on ice until storage at 4 °C. Unfiltered water was also collected at each interval for analyzing total trace metals and cations (acidified with 1–10% SEASTAR™ BASELINE**®** HNO_3_ in trace-metal-free 50 mL falcon tubes). Total and filtered (< 0.22 µm) iron concentrations were collected only in October 2020. All samples were transported within 24 h to the respective storage conditions. From 2019 to 2021, unfiltered water samples were collected at different periods of stratification for plutonium concentrations and isotope analyses (1 L stored in trace-metal-free bottle).

One sediment core at the Pond B inlet was collected in August 2020 using 30 cm polycarbonate tubes (inner diameter 5 cm) contained within an aluminum core sediment sampler. The core was transported on dry ice until storage at − 80 °C within 24 h. In May 2021, several surface sediment samples (~ 5 cm deep) were collected from the R Reactor canal, Pond A and B Canal (Fig. 1a) for plutonium concentration and isotope ratio measurements. Within two hours of collection, samples were dried at 45 °C for 48 h and then combusted within a muffle furnace at 900 °C for 2 h using 20 mL porcelain crucibles and stored for additional digestion and purification.

### Geochemical measurements

#### Total organic carbon, trace elements, and anion measurements

Along with the Sonde field data, geochemical analyses were performed in the lab, including TOC (< 0.22 µm filtered), major anions (filtered), and trace metals and major cations (unfiltered and acidified) (Supplementary Fig. 2). TOC was analyzed on a Shimadzu TOC analyzer (SSM-5000A) with default settings. Major anions (F^–^, Cl^–^, Br^–^, NO_3_^–^, NO_2_^–^, SO_4_^2–^ and PO_4_^3–^) were analyzed by following EPA Method 300.0 on a Metrohm 881 Compact Ion Chromatograph Pro with ProfIC Detector MF and a Metrosep A Supp 5–250 High Resolution Anion Column with Metrosep RP2 Guard. Major cations and trace metals (As, Ca, Co, Fe, K, Mg, Mn, Na, U, and Th) were analyzed following the EPA Method 200.8 on a Thermo Electron iCAPQ Inductively Coupled Plasma Mass Spectrometer with a quadrupole mass analyzer (QICP-MS).

#### Radionuclide measurements

All radionuclide measurements were conducted at Lawrence Livermore National Laboratory. Cesium-137 activities from one-liter unfiltered water samples were measured by gamma spectrometry using six different high-purity germanium detectors. A one-liter bottle spiked with 12.1 Bq ^137^Cs of NIST SRM 4233C-37 was used as a cross-calibration standard.

One liter unfiltered water samples, used for plutonium concentration and isotope ratio measurements, were digested with concentrated nitric acid (HNO_3_, SEASTAR™ BASELINE**®** Chemicals) and hydrogen peroxide (H_2_O_2_, SEASTAR™ BASELINE**®** Chemicals) at room temperature for at least 24 h prior to purification. A high purity ^244^Pu spike was added to the sample along with a ferric chloride standard and allowed to equilibrate for 12 h. Ammonium hydroxide (ACS grade) was gradually added to the sample to precipitate ferric hydroxides and co-precipitate plutonium. The solid precipitate was separated from the supernatant using centrifugation and digested with HNO_3_ at 110 °C. A process blank containing a low-ionic matrix similar to Pond B water was also purified in the aforementioned procedure for every batch of samples (Supplementary Information). For solid samples, approximately 100–500 mg per sample of dried sediment from R canal, Pond A, B canal, or Pond B was ashed at 900 °C prior to digestion with HNO_3_ and hydrofluoric acid (HF) and ^244^Pu spike.

The plutonium purification procedure to remove the various matrix and isobaric interferences (e.g. uranium, americium) is detailed in Balboni et al. (2022)^2^. Briefly, the digested plutonium sample was reduced to Pu(IV) with supersaturated NaNO_2_ in 8 M HNO_3_ prior to loading on a conditioned 2 mL AG1 × 8 (100–200 mesh, Bio-Rad resin bed). The column was subsequently rinsed with 3 mL of 8 M HNO_3_ and 6 mL 9 M HCl. Plutonium was eluted with a mixture of 9 M HCl and hydroiodic acid (HI) by reducing Pu(IV) to Pu(III). The sample aliquot was dried and redissolved in 4 M HNO_3_ and supersaturated NaNO_2_ and then applied to a column containing 0.6 mL TEVA resin (100–150 mesh, Eichrom) for further separation of uranium from plutonium. After loading, the second column was rinsed with 2 mL 4 M HNO_3_, and 0.5 mL 9 M HCl. Two mL of 0.1 M HCl + 0.005 M HF and 6 mL of a 12:1 mixture of 9 M HCl: HI were added to elute plutonium from the column. Finally, the sample was purified through a smaller version (1 mL resin bed) of the first anion column before measurement by MC-ICP-MS.

Plutonium isotope abundances were measured on a Nu Plasma 2 (Nu Instruments) with an Aridus II desolvating nebulizer (Cetac Technologies). Five full-size secondary electron multiplier ion counters monitored ^244^Pu, ^242^Pu, ^241^Pu, ^240^Pu, and ^239^Pu, while mass 238 was measured using a Faraday detector. The CRM 137 spiked with ^242^Pu was used to correct for instrumental mass bias and detector gain corrections with a dynamic, three-cycle multi-ion counting routine measuring ^239^Pu, ^240^Pu and ^242^Pu. Additional standards, CRM 126-A, 136, and 138, were measured twice each analysis run for quality control. Isotope dilution using the ^244^Pu spike allowed for quantification of ^242^Pu, ^241^Pu, ^240^Pu, and ^239^Pu in each sample. Typical ^244^Pu spike recoveries ranged from 60 to 80%. One unspiked sample was also measured to confirm that no ^244^Pu from other SRS activities was present in the samples^21^. Uncertainties reported represent twice the standard uncertainty of the analytical measurement. Positive detection of isotopes was considered significant if signals were greater than three times the standard uncertainty of the measurement. The average plutonium in procedural process blanks was < 0.5 fg or < 0.03 fCi ^239^Pu (n = 10).

### Data analysis

Relationships between the geochemical parameters of the water samples for the March and June sampling campaigns were analyzed using principal component analysis (PCA) with FactorMineR^24^. Variables not measured for specific samples were predicted using the imputation expectation–maximization algorithm within the R package, zCompositions. Highly collinear variables with Spearman coefficient (ρ^2^) greater than 0.6 were then excluded.

Thermodynamic calculations of plutonium and iron species in the water column were determined using Geochemist’s Workbench. Iron speciation referenced the PHREEQC Database^25^, while calculations for plutonium utilized the National Energy Agency Thermochemical Database^26^. In order to simulate amorphous Fe(III) mineral phases, both hematite and goethite were suppressed from formation.

## Results and discussion

### Effects of stratification on water column temperature, DO, pH, TOC, and iron

**Figure 2:**
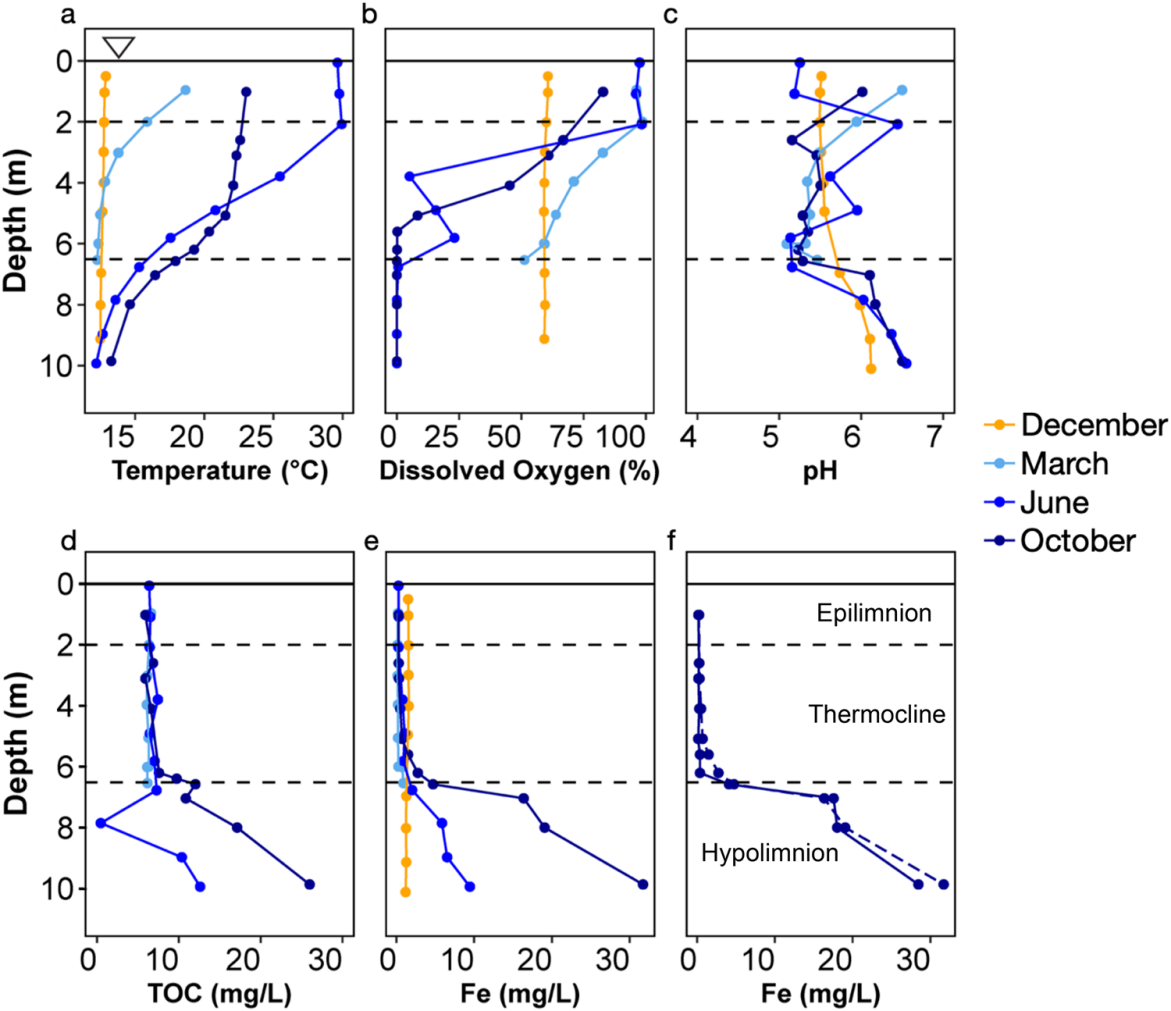
Water column profiles of (**a**) temperature, (**b**) dissolved oxygen (% saturation), (**c**) pH, (**d**) total organic carbon, (**e**) total iron, and (**f**) filtered iron (< 0.22 μm filter) from the Middle location. Samples from the December and March campaigns represent the unstratified period, while those in June and October were taken during stratification. The extent of the thermocline is indicated by the two dashed horizontal lines. Uncertainties represent the standard error of the measurement.

The Pond B water column chemistry is highly influenced by the monomictic nature of the pond. This is most apparent in the water column profiles of temperature and redox during stratification (June, October) (Fig. 2a,b). During summer stratification in June, temperature decreased from greater than 28 °C between 0 and 2.5 m depth to an average of 13.8 °C below 6 m. Dissolved oxygen similarly declined from greater than 97% saturation in the first 2 m to less than 1% below 6.5 m. The October data show a similar decreasing trend. During this sampling campaign, dissolved oxygen also decreased sharply at ~ 2.0 m at the Inlet-Middle, and Outlet locations to < 10% at 2.9–4.4 m confirming that the changes in temperature and redox conditions of the water column are strongly controlled by the stratification of the pond (Fig. 2**, **Supplementary Fig. 1). At 5.5 m, a moderate peak in dissolved oxygen was evident at the Middle and Inlet-Middle location before dropping to < 0.5% below 6 m (Fig. 2**, **Supplementary Fig. 1). This peak coincides with a chlorophyll A peak at ~ 6.5 m^17^, indicating microbial oxygen-producers reside at this depth. In October, both the temperature and dissolved oxygen gradient were more muted than in June, declining from 23 °C at 1 m depth to 13 °C at 10 m. In contrast to the June data, dissolved oxygen dropped more in October, from greater than 80% at the shallowest depth to < 1% below 5.5 m, likely reflecting the ecological succession of more anaerobes in the later months of stratification^17^.

During the unstratified sampling campaigns (December and March), the Pond B water column was more homogeneous with respect to temperature and dissolved oxygen profiles (Fig. 2, Supplementary Fig. 2). In December, both the temperature and dissolved oxygen are completely homogenous, most likely as a result of full mixing of the water column during turnover. In contrast, in March, the surface is fully oxygenated, but there is a slow and gradual decline of dissolved oxygen in the thermocline. Along with a similar temperature decrease, this would suggest that stratification is just beginning to occur during March.

Consistent with previous studies^23^, the stratified and unstratified water columns at the Middle location were similarly acidic (stratified average pH: 5.75, unstratified average pH: 5.72), but have a large range with respect to depth (4.56–6.84). In the June and October stratified water column, the local shifts in pH were present throughout the thermocline (Fig. 2). The pH is lower (~ 5.20) at the base of the thermocline. Below this depth, there is a sharp increase in the hypolimnion to pH 6.80 at the sediment–water interface, which is more favorable for Fe(III) reduction. This is in marked contrast to the gradual increase in pH observed in deeper waters within the unstratified samples (pH 5.50–6.12).

**Figure 3:**
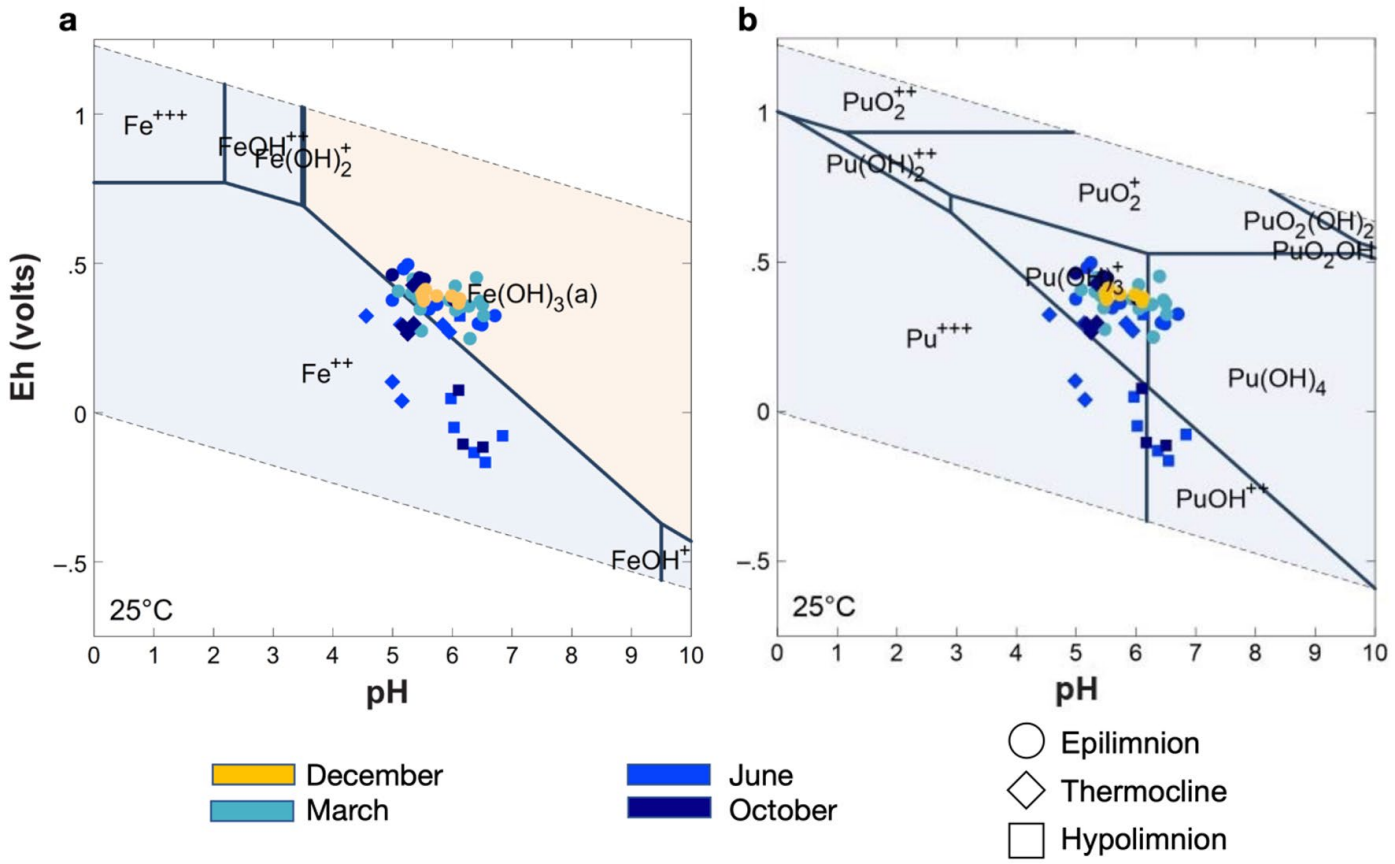
Distribution of relevant (**a**) iron and (**b**) plutonium aqueous (light blue field) and solid (orange field) species in Pond B water by Eh and pH. Species distribution was modeled with Geochemist’s Workbench^43^ with [Fe]:10^–5^ and [Pu]:10^–16^.

Reduction and dissolution of Pond B sediments containing Fe(III)-(oxyhydr)oxides drives the release of Fe^2+^ and organic matter, similar to other studies of seasonally stratified lakes^16^. In the December and March water column, iron and TOC do not vary by depth or location (Fig. 2). In contrast, stratification results in an increase in TOC and total iron in the deeper waters below the thermocline in June and October. Figure 3a is a plot of the expected iron species and shows that solid Fe(III) phases were dominant in the epilimnion of the stratified periods and the entire unstratified water column, while Fe^2+^ is present in anoxic waters during stratification. In December, approximately one month after turnover and pond mixing, iron concentrations and TOC remained low throughout the water column, likely because oxidized Fe(III) complexes with particulate organic matter (POM) and Fe-POM aggregates settle to the oxygenated bottom of the pond. During stratification in the summer and early fall months, pH, TOC, total dissolved solids (TDS) and iron concentrations increase in the deepest locations of the hypolimnion, where dissolved oxygen is less than < 0.5% (Fig. 2, Supplementary Fig. 1 and 2). Greater than 90% of the total iron was less than 0.22 μm or Fe^2+^, which would be thermodynamically favorable in the deeper thermocline and hypolimnion layers. Possible mechanisms driving the release of Fe^2+^ and TOC from the sediments include microbial Fe(III) reduction and organic matter degradation in the hypolimnion and shallow sediment cores^17^. In October, iron concentrations increase in the hypolimnion relative to June (Fig. 2). Increases in the abundance of anaerobic microorganisms in the hypolimnion likely drive the deeper parts of the pond to more reducing conditions during the later periods of stratification^17^.

### Plutonium concentrations are highest in spring and in deep waters during stratification

**Figure 4:**
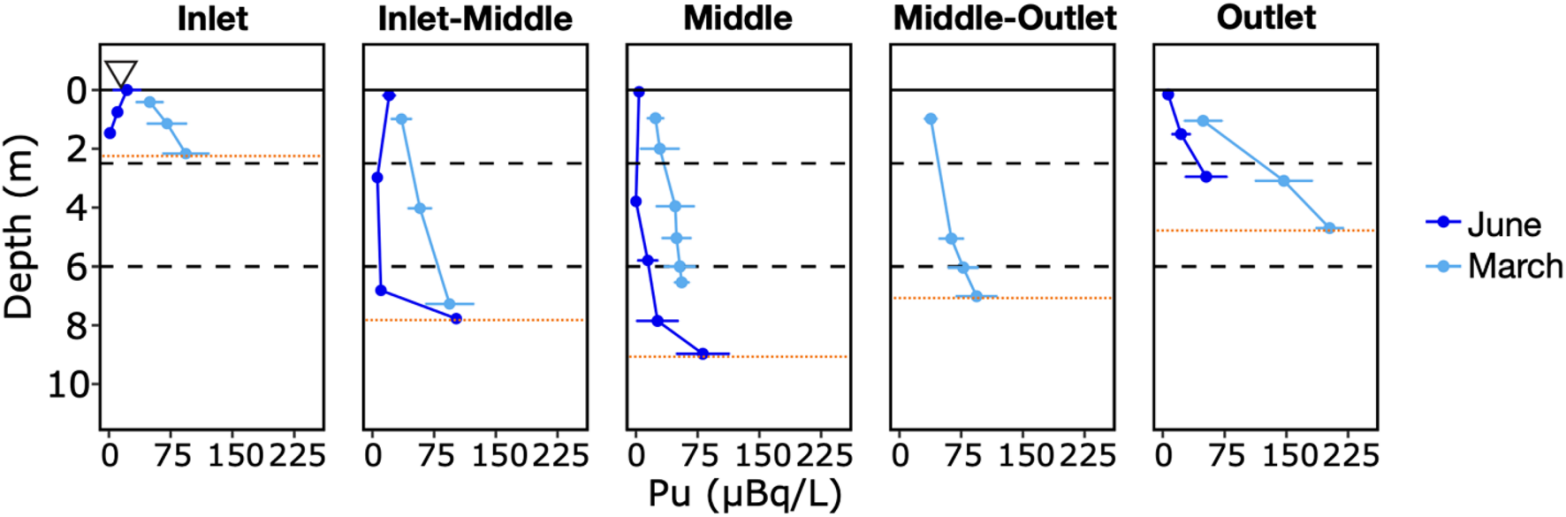
Water column profiles of plutonium at all five locations across transect A to A’, measured when Pond B was unstratified for 4 months (March, light blue) and stratified for 3 months (June, dark blue). Uncertainties represent twice the standard uncertainty for each measurement. Dashed horizontal lines indicate extent of the thermocline. Maximum depths (red horizontal lines) at each location are as follows, Inlet: 2.1 m, Inlet-Middle: 7.3 m, Middle: 9.8 m, Middle-Outlet: 8.3 m, and Outlet: 4.7 m.

Plutonium concentrations (Fig. 4) at all five locations showed less variability between stratified and unstratified periods compared to dissolved oxygen or iron concentrations (Fig. 2). In all water samples, the plutonium concentrations are greater in the unstratified samples (December, February, March) relative to the stratified samples (June, October). In addition, plutonium concentrations in the unstratified water column increased gradually with depth to varying degrees, with the outlet displaying the most elevated concentrations at a maximum of 202.6 μBq L^−1^ (~ 5 m deep). Overall, plutonium concentrations increase close to the sediment–water interface (Fig. 4), suggesting that plutonium remobilization occurs at this boundary even at shallow depths. During the period of pond stratification, concentrations of plutonium in the epilimnion and thermocline were consistently lower than the hypolimnion in the Inlet-Middle and Middle locations.

**Figure 5:**
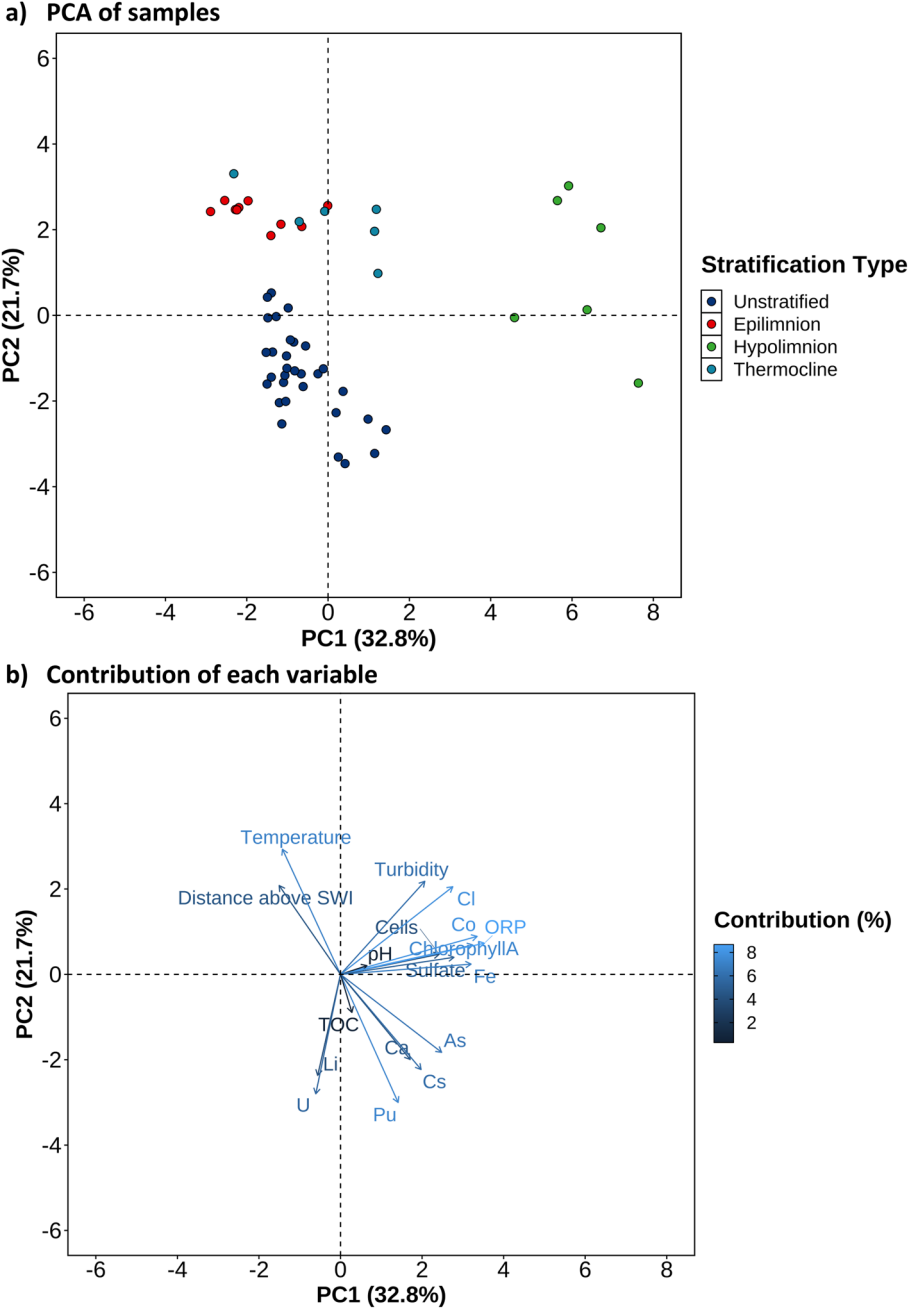
Principal component analysis (PCA) of the physical and geochemical data. (**a**) Variation of samples by stratification type: unstratified (March) and stratified (epilimnion, thermocline, hypolimnion; June). (**b**) Contributions of each variable. SWI = sediment–water interface, ORP = oxidation–reduction potential, TOC = total organic carbon.

To understand the plutonium cycle in Pond B, principal component analysis (PCA) was performed on the water column’s physical and geochemical properties (Fig. 5). In our analysis, the first three dimensions explain 65.2% of the variance (1: 32.8%, 2: 21.7%, 3: 10.7%) (Supplementary Table 1), and there is a clear separation between the stratified and unstratified sampling campaigns (Fig. 5a). In contrast, there is no apparent relationship among different locations in the Pond B transect (Supplementary Fig. 6), indicating stratification is a more influential factor on water column chemistry than location Fig. 5); this is also reflected in the microbial community composition^17^. For Principal Component 1 (PC1), the top contributing variables (> 10%) are ORP, cobalt, chlorophyll, and iron; and for PC2, the major variables are plutonium, temperature, and uranium (Fig. 5b, Supplementary Fig. 4 and 5). The unstratified samples are spread along PC2, and as discussed above, plutonium concentrations are highest in the unstratified water column. The plutonium vector is also almost orthogonal to ORP, iron, and other variables that are more associated with the hypolimnetic samples.

The mechanisms for plutonium release from shallow, oxygenated Pond B sediments (i.e., Inlet and Outlet) remain unclear. Coutelot et al., (2022)^4^ observed accumulation of plutonium in organic matter rich sediments near the Pond B outlet indicating a strong association between plutonium and iron/carbon cycling. A comparable re-distribution was not observed for Cs-137 which has a relatively lower affinity for organic matter than plutonium^27^. Furthermore, production of Fe-POM stable colloids is known to occur at redox interfaces in organic rich environments^28^. This may explain the increase in plutonium concentrations in the outlet water column relative to the inlet (Fig. 4), despite greater concentrations of radionuclides in the inlet sediments. The lower depths of the outlet water column had low dissolved oxygen concentrations (less than ~ 25%) during unstratified and stratified time periods (Supplementary Fig. 1), and outlet sediments contained three-fold as much organic carbon relative to the inlet sediments^4^. Higher relative abundances of putative organic matter degraders were also observed in the outlet compared to the inlet sediment cores; these degraders could promote release of colloids from the sediments^17^. Physical resuspension of Fe–Pu-POM from storm-induced wave action or underwater currents present during holomixis has also been suggested as another pathway for remobilization^14^. In addition, the absence of large aquatic macrophytes, which may act like baffles for Fe–Pu-POM, in shallow parts of the pond during less productive months may also increase particulate remobilization in the pond. Overall, these possible mechanisms suggest that multiple processes may likely contribute to plutonium release from shallow sediments.

Based on geochemical modeling of solution conditions, plutonium in the Pond B water column likely exists either in the + 4 or + 3 oxidation states (Fig. 3). The Pu(III) oxidation state species are more soluble than Pu(IV) species^29,30^. In oxygenated, relatively low organic matter waters, Pu(IV) likely predominates, primarily as Pu(OH)_3_^+^ and at higher pH, Pu(OH)_4_ but concentrations are profoundly limited by formation of PuO_2_(s). In more reducing waters, Pu^3+^ and PuOH^2+^ are favored. Data on plutonium complexation with natural organic matter are relatively limited but indicate that plutonium solubility will be greatly increased in the presence of natural organic matter^31–33^. Reductive dissolution of iron-rich Pond B shallow sediments coupled with the reduction of Pu(IV) results in the diffusion of aqueous Pu^3+^ or plutonium complexed to organic matter to the more oxygenated thermocline. Alberts et al. (1987)^13^ first proposed this seasonal reduction of Pond B sediments as a mechanism for plutonium remobilization. However, the relatively weak correlation between plutonium and redox conditions in the water column (based on our PCA analysis) suggests that plutonium release from sediments because of reductive dissolution during stratification may not be the dominant mechanism for seasonal plutonium remobilization in Pond B. Instead, processes driving plutonium release from the sediments likely include a combination of reductive dissolution, sediment resuspension, and microbial activity which leads to increases in Fe-POM and stabilizes plutonium in the pond water column.

It is likely that plutonium within the fully oxidized water column is transported in the particulate or colloidal phase associated with Fe(III) phases and POM, as observed in a variety of other environments^34–36^. As reported in numerous previous studies, interactions with organic matter and Fe(III) (oxyhydr)oxides can greatly influence plutonium mobility. Plutonium complexes with carboxyl and nitrogen groups in natural organic matter^37^. The presence of natural organic matter can increase the adsorption of Pu(IV) to Fe(III) solid phases at lower pH^38,39^. Plutonium complexation to organic matter may be driven directly or indirectly through microbially-driven processes, such as sorption to cells and POM aggregates, bioaccumulation of plutonium, and complexation by microbial products; this is further discussed in Part II^17^.

### Plutonium in pond B is isotopically similar to upstream canal sediments

**Figure 6:**
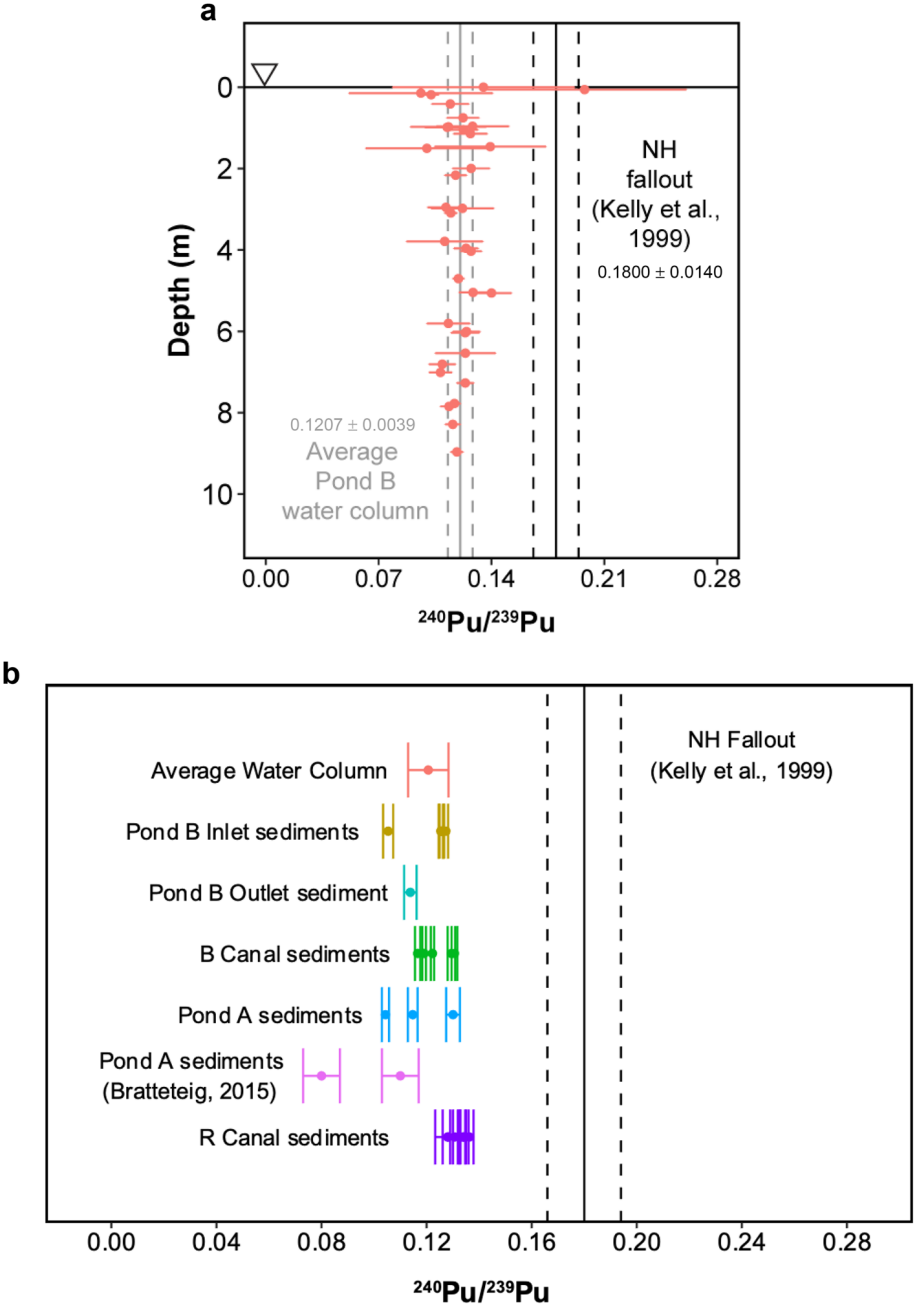
(**a**) Plutonium isotope ratios for water column samples by depth. Error bars represent twice the standard uncertainty on an individual measurement. Sample points overlap with average water column values (grey solid and dashed lines), but are mostly distinct from Northern Hemisphere fallout (black solid and dashed lines). (**b**) Compiled plutonium isotope ratios for sediment^41^ and water samples.

Plutonium isotope ratios in the water column were primarily constant among location, depth, and sampling campaign (Fig. 6a); the average stratified and unstratified ^240^Pu/^239^Pu measuring 0.1207 ± 0.0077 and 0.1210 ± 0.0025, respectively. All water column isotope ratios were indistinguishable from this average except one epilimnion sample in the Middle location (0.1977 ± 0.0632), which overlapped with Northern Hemisphere fallout values (0.1800 ± 0.014)^40^. The overall isotopic homogeneity indicates that plutonium originates from a distinct source, which still overwhelms the fallout-derived plutonium about 60 years after being released into the pond.

Plutonium concentrations in the sediments are highest in the inlet core of Pond B followed by the canal between R Reactor and Pond A (Supplementary Table 2). Sediment samples from all the nearby locations show a degree of isotopic variability (σ: 0.009). However, the average of all of the sediments measured in this study (0.1204 ± 0.0038) is indistinguishable from the average ^240^Pu/^239^Pu of water column samples (0.1207 ± 0.0077) and significantly lower than Northern Hemisphere fallout. Plutonium isotopic measurements reported previously from Pond A (0.08 ± 0.01 to 0.11 ± 0.01)^41^ were lower than all samples measured.

While there are no historical records of ^240^Pu/^239^Pu released from R Reactor, the similarity between upstream isotope ratios of the shallow sediments and the average water column values suggest R Reactor releases are the predominant source of plutonium in Pond B. Previous isotopic measurements by Bratteteig (2015)^41^ of an 18 cm core sampled from Pond A display variability by depth. The average of the top 7 cm ^240^Pu/^239^Pu are significantly higher than the deeper 10 cm (Fig. 6b). We also observe a similar trend in the Pond B Inlet core, where the first 4 cm below the sediment water interface had isotopically high values compared to a sample at 8.6 cm depth (0.1054 ± 0.002) (Supplementary Table 2). Lower ^240^Pu/^239^Pu in deeper sediments hints at a history of multiple isotopically distinct plutonium releases that impacted Pond A and Pond B. The isotopically lower values also correlate with lower plutonium concentrations in the Inlet core (Supplementary Table 2). This may be associated with a smaller release prior to the documented instance in 1957 of 1.11 × 10^10^ Bq of plutonium, and the small spread in ^240^Pu/^239^Pu within B Canal, Pond A, and R Canal could be the result of mixing between more than one release.

### Pond B plutonium concentrations have decreased over the last 35 years

**Figure 7:**
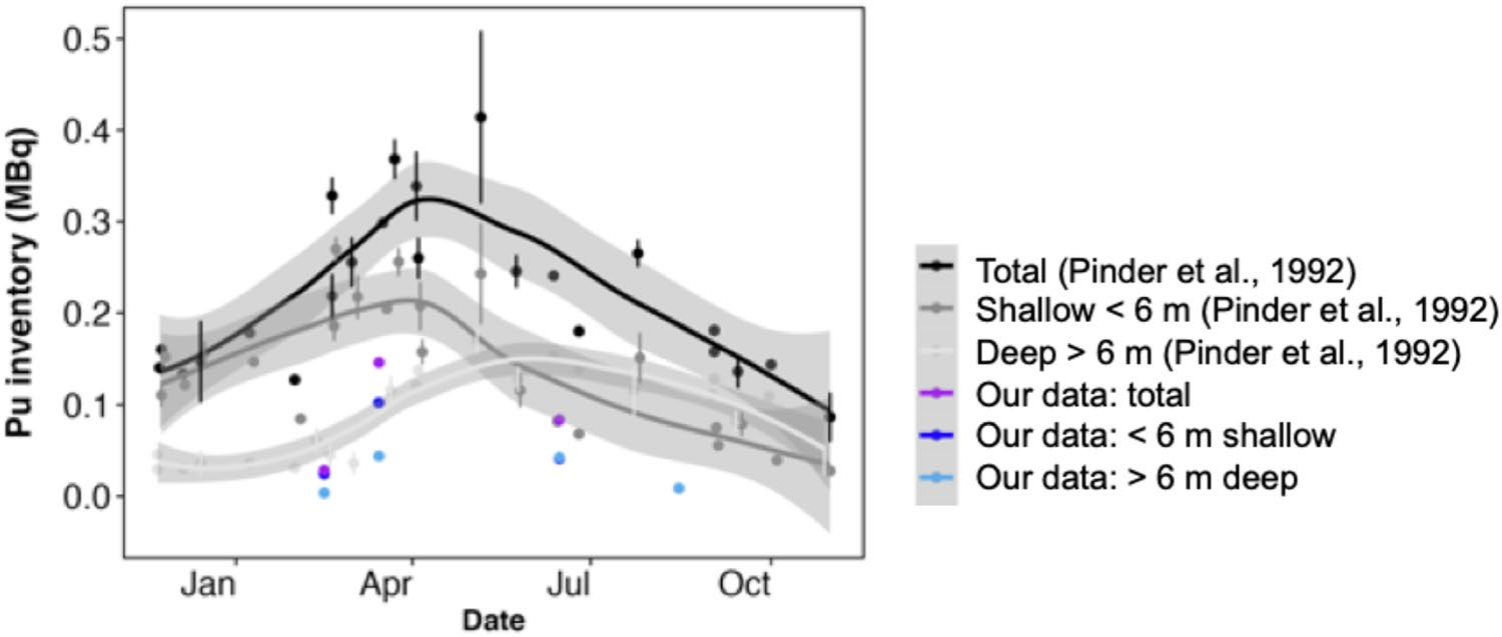
Plutonium inventories in the Pond B water column calculated from Pinder et al. (1992)^14^ and our data. The grey envelope signifies the smoothing function using the LOESS regression for the data from Pinder et al. (1992)^14^.

Total inventories of plutonium in Pond B water in this study are a factor of 2–2.5 lower than inventories calculated from data collected in 1984^14^. However, the relative contributions from deep and shallow inventories reported in Pinder et al. (1992)^14^ are similar to our observations. For example, the total plutonium in the water peaked in early spring (March–April) and declined over the summer (Fig. 7). In addition, the shallow water column (< 6 m) in Pond B contributes the most plutonium to the total inventory in February and March, while the deeper inventories were higher or equal to shallow inventories in June and August.

In Pond B, plutonium largely remains immobilized in the sediments, as demonstrated by the isotopic evidence and profiles of plutonium in the water column and sediment. Coutelot et al. (2022)^4^ noted a sharp peak of ^239^Pu present within the first 5 cm depth of a core sampled from the inlet. Integrated concentrations of ^239^Pu (3.8 × 10^5^ Bq m^-2^) relative to total plutonium in the inlet water column (0.15 Bq m^-2^) indicate a partitioning ratio of plutonium between the overlying pond water and sediments to be 4 × 10^–7^. At the outlet, the ratio is higher (1.3 × 10^–4^). Nevertheless, plutonium is retained in both the inlet and outlet sediments.

As discussed previously, the outlet contains higher organic matter content relative to the inlet^4^. As organic matter complexation and particulate transport may remobilize plutonium in shallow waters and the unstratified water column, the outlet sediments could represent a preferential sink for plutonium in Pond B that limits plutonium migration out of the pond. However, the integrated plutonium mass in the outlet sediments is still very small relative to the inlet sediments^4^, implying that plutonium immobilized in the outlet sediments represents only a small fraction of the plutonium inventory in the pond. Given the pond turnover rate of ~ 3 years proposed by Whicker et al. (1990)^21^ and the small integrated fraction of plutonium in pond water relative to sediments, plutonium should remain in the sediments for an interminable period of time.

Decreasing rates of plutonium release from Pond B sediments over time may be responsible for the two to three-fold decrease in plutonium water concentrations since the mid-1980s. The decline of plutonium concentrations relative to previous data is more pronounced in deeper waters (> 6 m) relative to shallow waters (0–6 m) (Fig. 7). As reductive dissolution of sediments is likely responsible for the majority of plutonium release in the hypolimnion (> 6.5 m) lower inventories of plutonium imply greater loss of this easily remobilized pool and/or increasing recalcitrance of plutonium in the deeper sediments. In the former mechanism, plutonium release during reductive dissolution from shallow sediments results in a fraction of this pool being exported from Pond B or redistributed to other sinks in the pond system instead of resettling to the bottom of the pond during holomixis. The second pathway of increasing recalcitrance is likely because of mineral and sediment aging effects, which have been documented to inhibit plutonium desorption^2,37,42^. Both Lin et al. (2019)^37^ and Balboni et al. (2022)^2^ observed immobilized fractions of plutonium in sediments (with depth) decades after deposition. This pool is associated with less biolabile organic matter associated with Fe(III)-(oxhydr)oxides, which acts as a physical barrier to organic matter degradation. These sediment aging effects may be responsible for lower release of the plutonium in the inlet shallow sediments. In deeper waters, increased crystallinity of Fe(III)-(oxyhydr)oxides driven by annual redox oscillations may also result in greater protection of Fe–Pu-POM complexes or even incorporation of plutonium into the solid phase by dissolution and re-precipitation processes.

## Conclusion

Plutonium released from R Reactor in the late 1950s to Pond B is largely immobilized in the pond’s sediments. The majority of plutonium within Pond B originates from documented R Reactor releases in the late 1950s as it is isotopically identical to shallow upstream sediments. The current release from the pond sediments to the water column is low compared to the total inventory within the sediments.

While Pond B maintains seasonal redox cycling of iron and other trace metals, the impact on plutonium mobility directly from reductive dissolution due to stratification is secondary relative to organic matter transport in early spring. This mechanism of POM cycling in shallow waters remains unclear and should be studied further. Once suspended in the water column, the Fe(III)-POM-Pu complexes within the water column may be controlled directly or indirectly by thermocline structure and in particular, microbial metabolisms within the water column^17^.

Finally, our comparison to previous water column data measurements from Pinder et al. (1992)^14^ indicates that plutonium may be less easily remobilized into the water column over time. While this increasing recalcitrance in the sediments may minimize ecosystem impacts in Pond B, effects of sediment disturbance such as dredging or increasing frequency of large storms could result in the resuspension and remobilization of plutonium in the future. In addition, warming temperatures driven by climate change may impact both the timing and duration of stratification and lead to organic matter degradation within the pond’s sediments, resulting in greater plutonium remobilization to the water column.

## Supplementary information

Process Blank composition, trace element concentrations at Middle location, Inlet, Inlet-Middle, Middle-Outlet, Outlet concentrations, Scree plot, factor contribution to PC1 and PC2, sediment plutonium concentrations and isotope ratios.

## Supplementary Information

Supplementary Information.

## Data Availability

Geochemical data can be found on ESS-DIVE at https://doi.org/10.15485/1910298.
