# Supplementary material for: Sources, seasonal cycling, and fate of plutonium in a seasonally stratified and radiologically contaminated pond
Source: Sci Rep. 2023 Jul 8;13:11046. doi: 10.1038/s41598-023-37276-w (PMC10329635; doi:10.1038/s41598-023-37276-w)
Supplement: Supplementary file 1 — Supplementary Information. [file 41598_2023_37276_MOESM1_ESM.pdf]

Supplementary Information

Sources, seasonal cycling, and fate of plutonium in a seasonally stratified and radiologically contaminated pond

Naomi L. Wasserman*[1], Nancy Merino[1], Fanny Coutelot[2,3], Dan Kaplan[4], Brian Powell[2,3,4], Annie B. Kersting[1], Mavrik Zavarin*[1]

[1] Physical and Life Sciences Directorate, Lawrence Livermore National Laboratory, Livermore, CA, 94550, United States
[2] Department of Environmental Engineering and Earth Sciences, Clemson University, Anderson, SC, 29625, United States
[3] Center for Nuclear Environmental Engineering and Science and Radioactive Waste Management, Clemson University, Anderson, SC, 29625, United States
[4] Savannah River National Laboratory, Aiken, SC, 29625, United States

Correspondence:
* Naomi Wasserman
Lawrence Livermore National Lab
7000 East Ave, L-231
Livermore, CA 94550
925-424-6491
wasserman3@llnl.gov

Nancy Merino
Lawrence Livermore National Lab
7000 East Ave, L-452
Livermore, CA 94550
925-424-7605
merino4@llnl.gov

Process Blank Information

Each one-liter synthetic Pond B water sample contained 2 g L^{-1} NaCl, 0.5 g L^{-1} Mg_2SO_4, and 0.4 g L-1 $CaCl_2$ to roughly match the ionic composition of the pond. The one-liter sample was then adjusted to pH 6 using HCl.

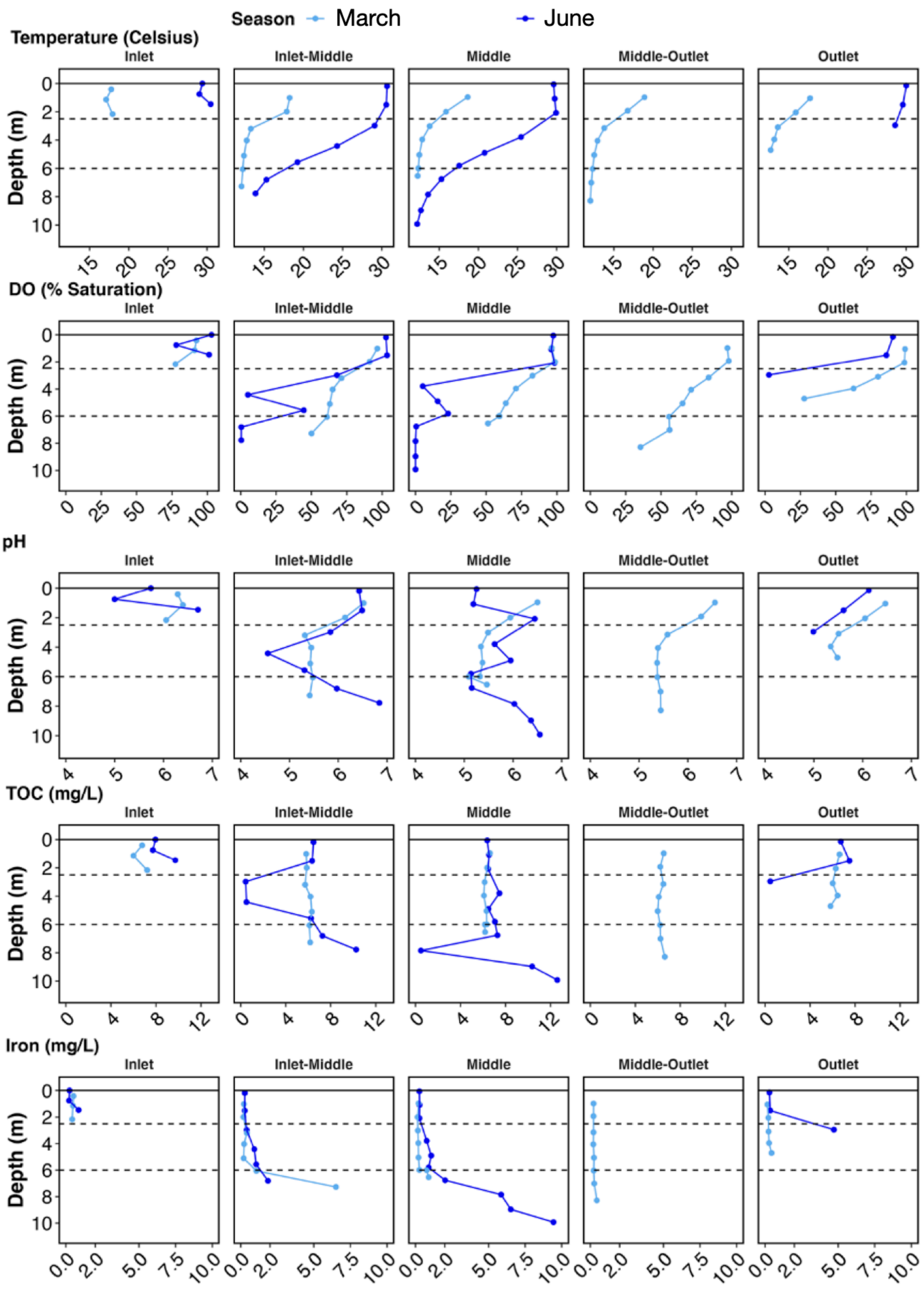

Supplementary Figure 1. Unstratified and stratified water column profiles for temperature, dissolved oxygen, pH, total organic carbon, and iron for all five locations.

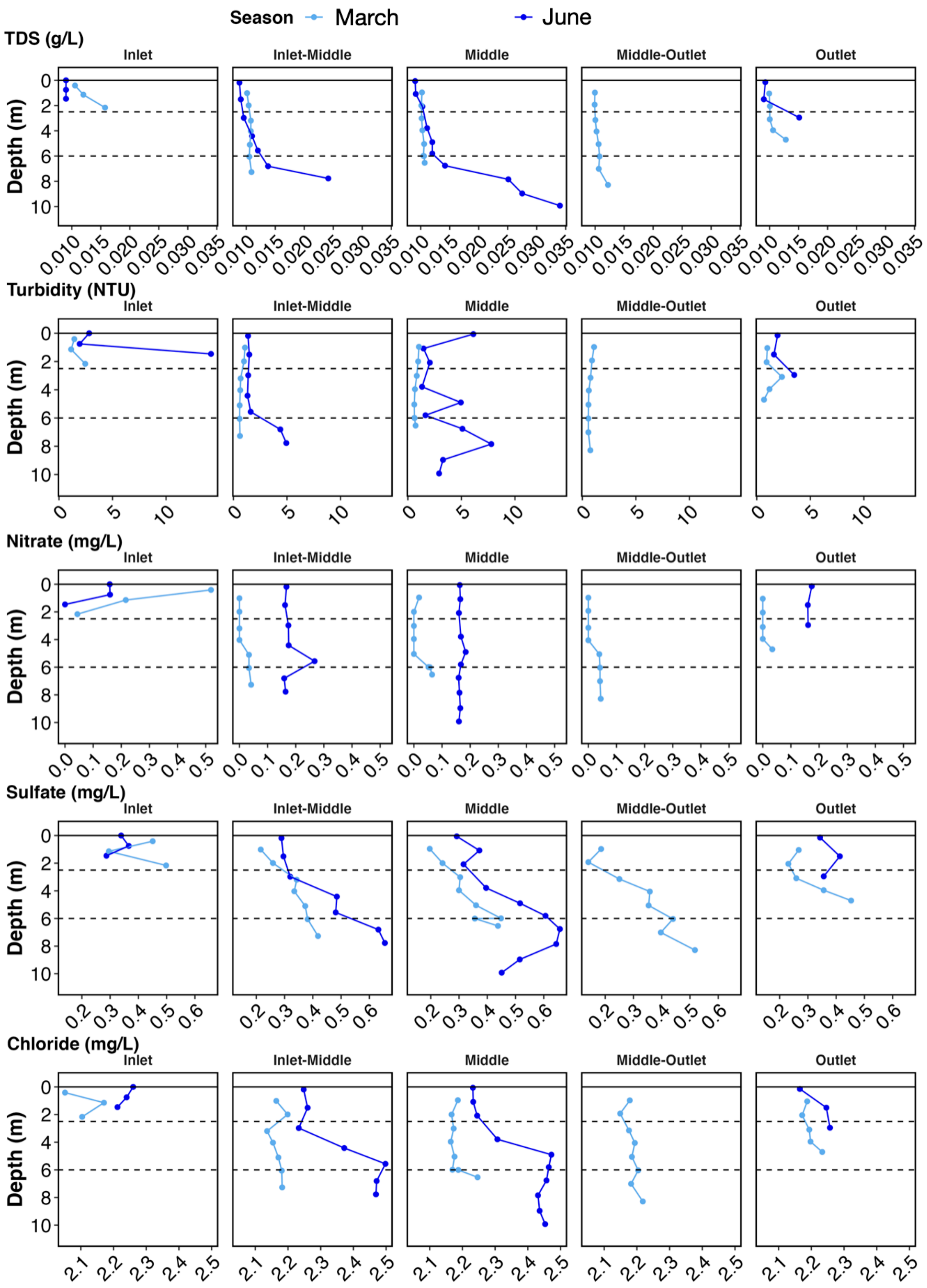

Supplementary Figure 2. Unstratified and stratified water column profiles for total dissolved solids, turbidity, nitrate, sulfate, and chloride for all five locations.

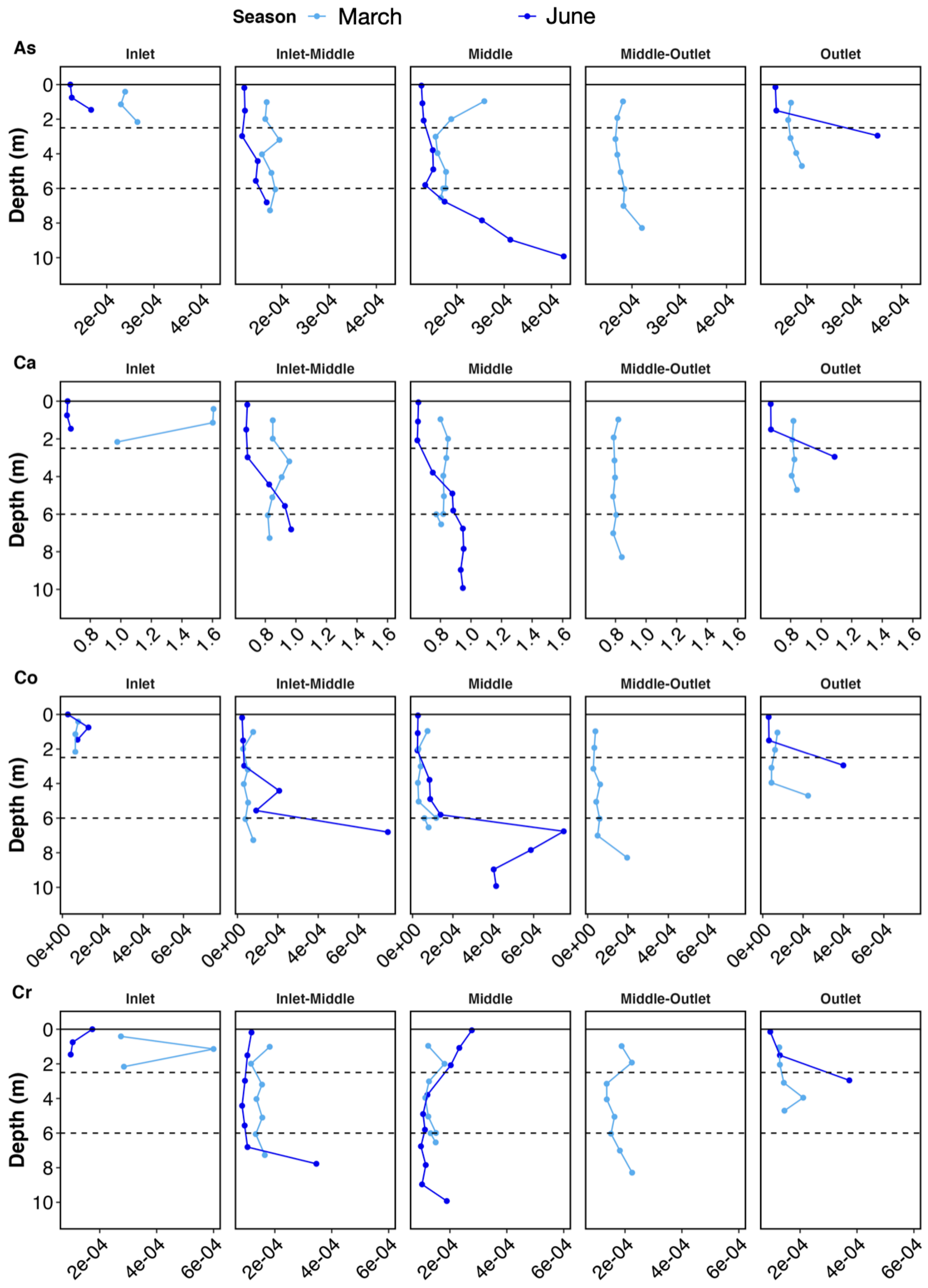

Season March June
As
Ca
Co
Cr
Inlet
Inlet-Middle
Middle
Middle-Outlet
Outlet
Depth (m)
0
2
4
6
8
10
2e-04
3e-04
4e-04
0.8
1.0
1.2
1.4
1.6
0e+00
6e-04

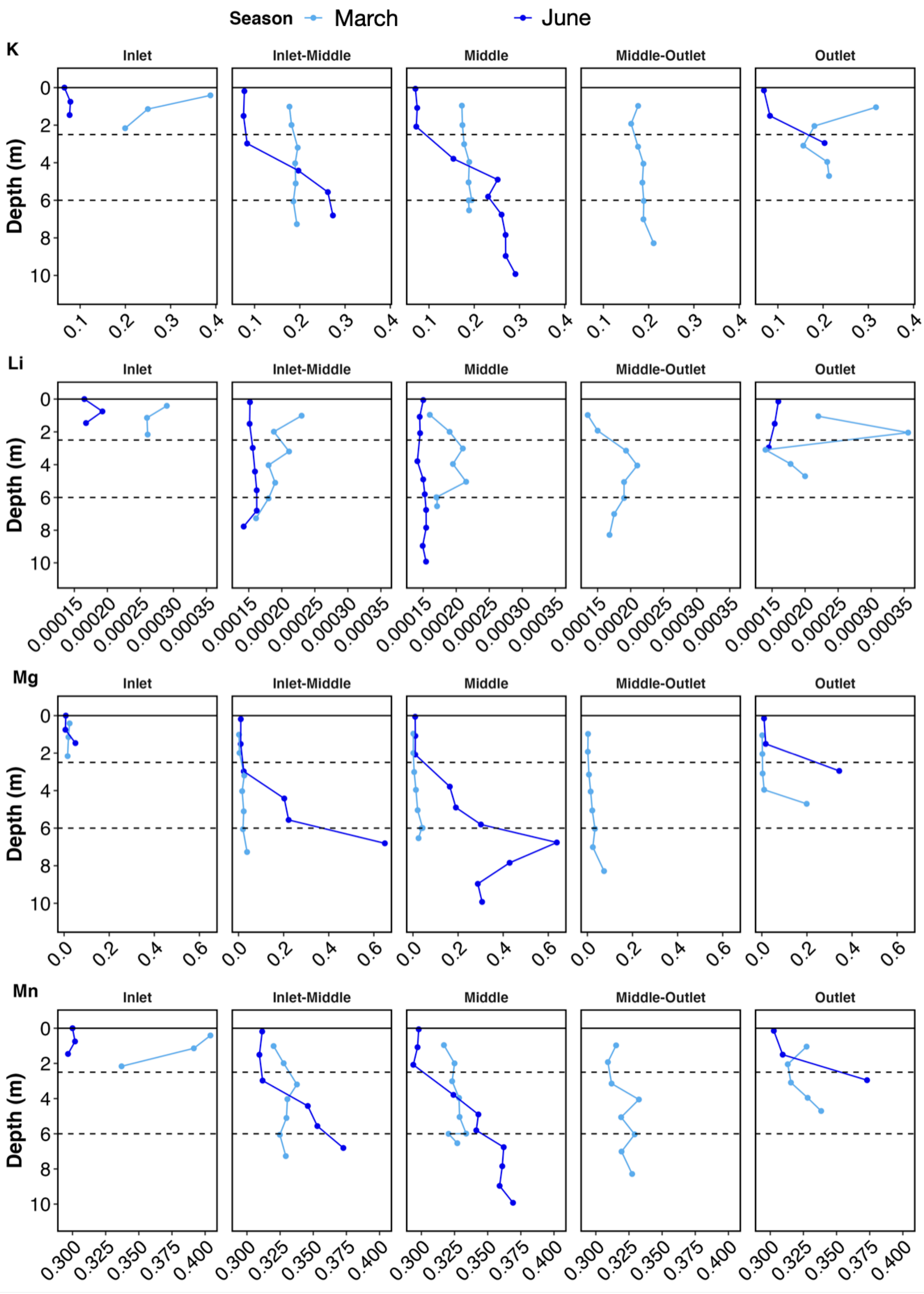

Season
March
June
K
Li
Mg
Mn
Inlet
Inlet-Middle
Middle
Middle-Outlet
Outlet
Depth (m)
0
2
4
6
8
10
0.1 0.2 0.3 0.4
0.00015 0.00020 0.00025 0.00030 0.00035
0.0 0.2 0.4 0.6
0.300 0.325 0.350 0.375 0.400

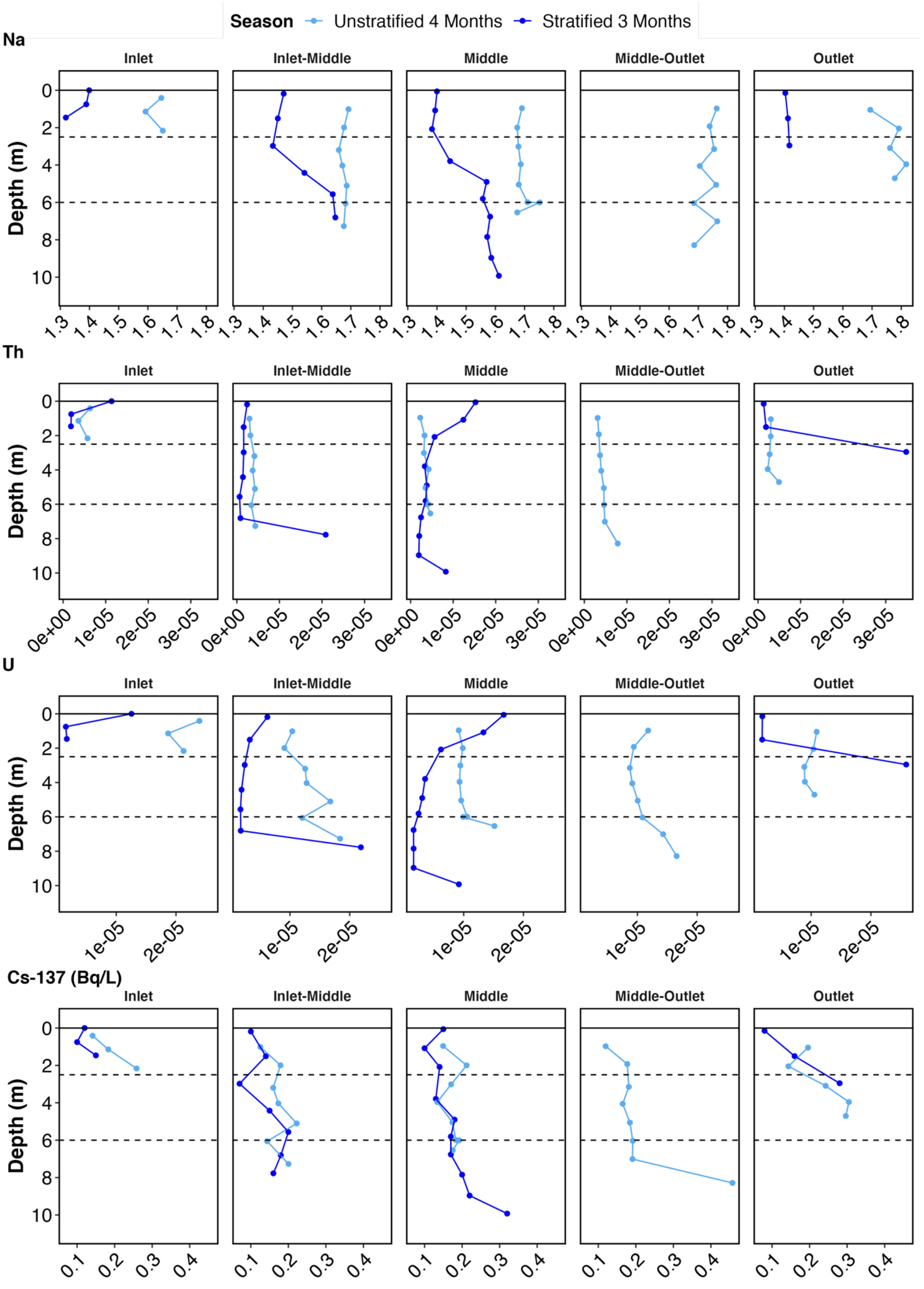

Season
Unstratified 4 Months
Stratified 3 Months
Na
Th
U
Cs-137 (Bq/L)
Inlet
Inlet-Middle
Middle
Middle-Outlet
Outlet
Depth (m)
0
2
4
6
8
10
1.3
1.4
1.5
1.6
1.7
1.8
0e+00
1e-05
2e-05
3e-05
0.1
0.2
0.3
0.4

Supplementary Figure 3. Unstratified and stratified water column profiles for metals in filtered and acidified (except for ^{137}Cs) fractions of the water for all five locations. All concentrations are in mg L^{-1}, with the exception of ^{37}Cs.

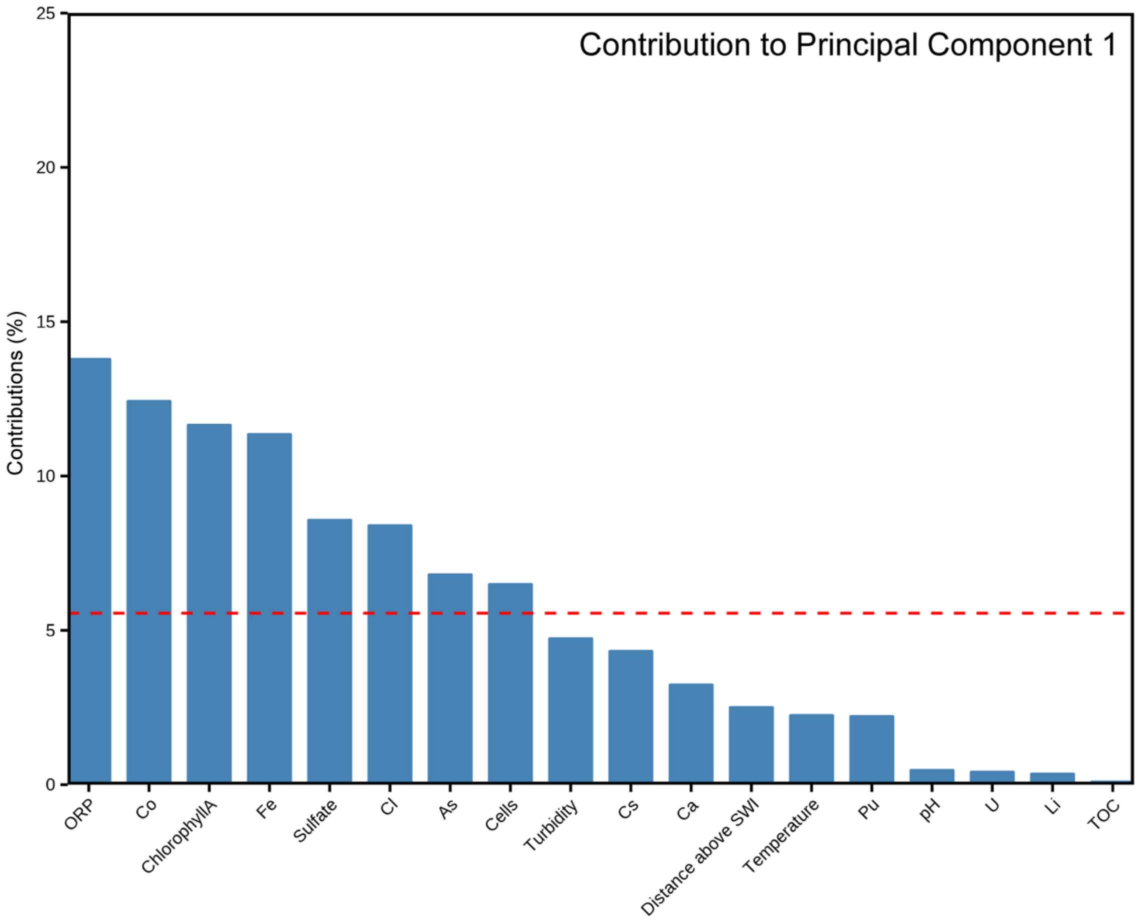

Supplementary Figure 4. Percent contribution of variables to principal component 1.

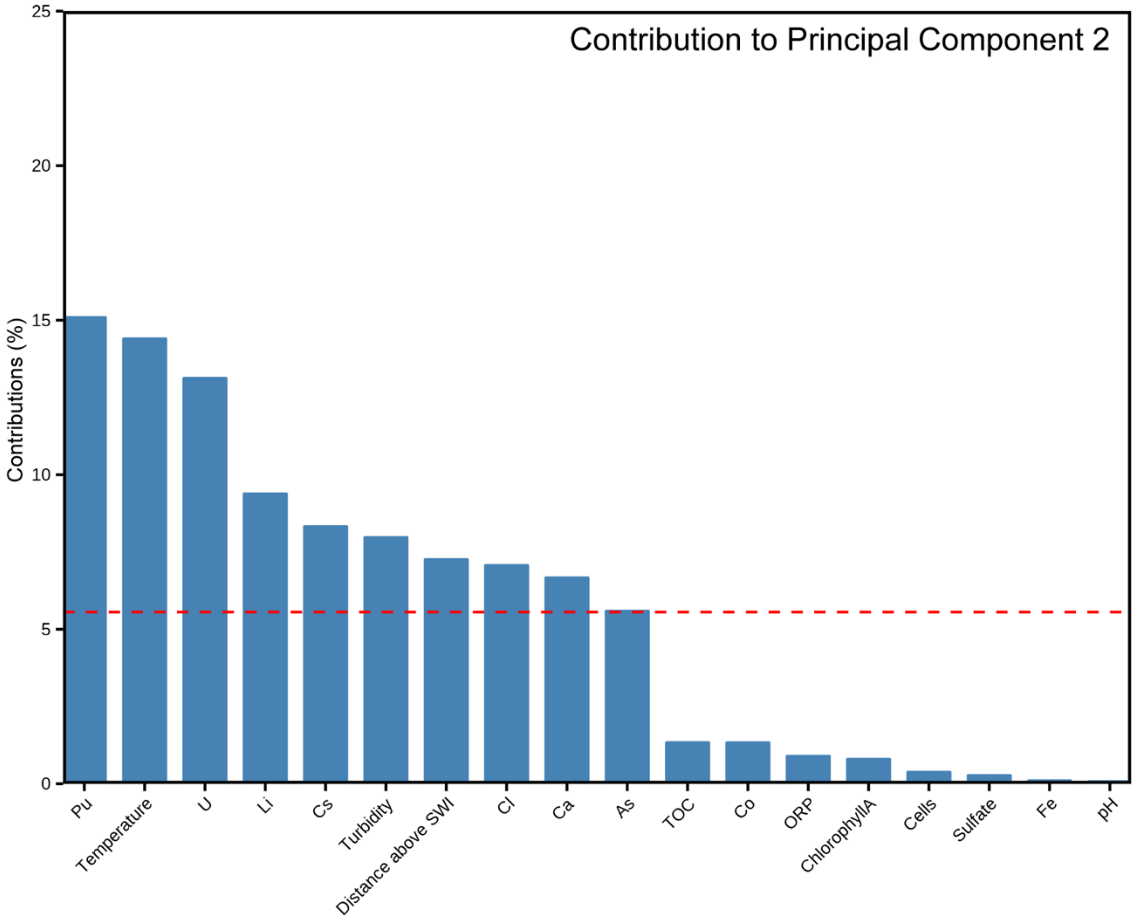

Supplementary Figure 5. Percent contribution of variables to principal component 2.

(a) Stratification Type
(b) Sampling Locations

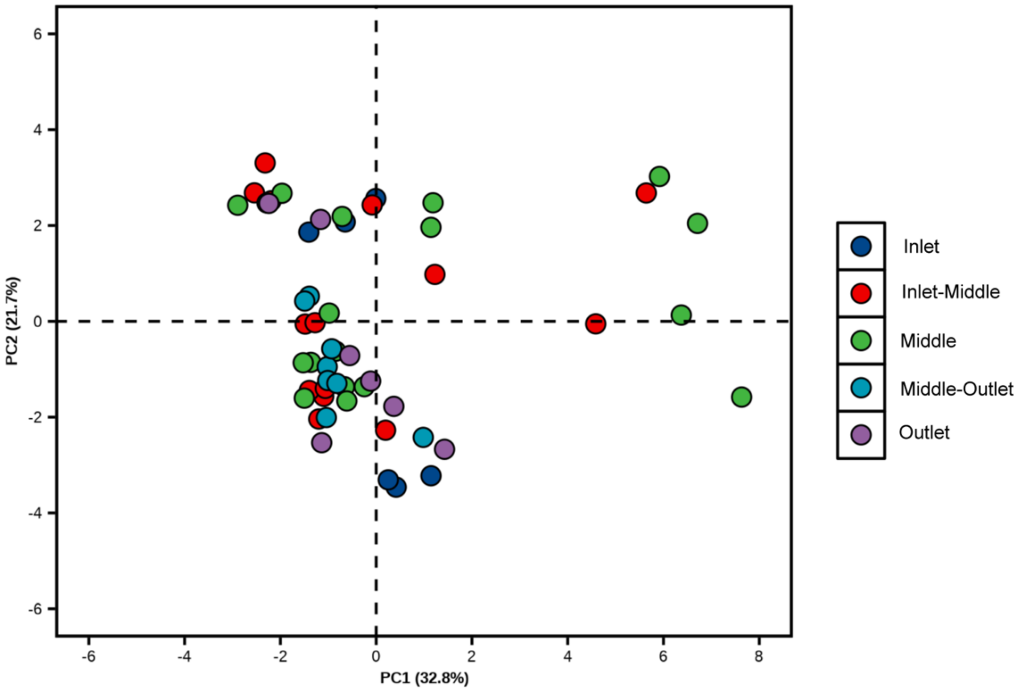

Supplementary Figure 6. PCA biplot of individual samples using principal components 1 and 2. Colors denote sample locations along the transect. All sampling campaigns are depicted.

Supplementary Table 1. Dimension eigenvalues and contribution to the overall variance for the principal component analysis.

Principal component	Eigenvalue	Variance (%)	Cumulative variance (%)
1	5.9	32.8	32.8
2	3.9	21.7	54.5
3	1.9	10.7	65.1
4	1.2	6.7	71.8
5	1.0	5.4	77.2
6	0.8	4.3	81.5
7	0.6	3.6	85.1
8	0.5	2.9	88.0
9	0.4	2.4	90.3
10	0.4	2.2	92.5
11	0.4	2.0	94.5
12	0.3	1.6	96.1
13	0.2	1.2	97.3
14	0.1	0.8	98.1
15	0.1	0.7	98.8
16	0.1	0.5	99.3
17	0.1	0.4	99.7
18	0.1	0.3	100.0

Supplementary Table 2. Sediment sample concentrations and associated isotope ratios.

Sample ID	Location	Depth (cm)	Concentration		Isotope ratio	
			mBq Pu g^{-1}	Standard uncertainty	$^{240}Pu/^{239}Pu$	Standard uncertainty
Core 1-2 #10	Inlet	8.60	0.36	0.01	0.1054	0.0019
Core 1-2 #5	Inlet	3.60	10.41	0.22	0.1257	0.0009
Core1-2 #4	Inlet	2.60	19.77	0.41	0.1258	0.0008
Core 1-2 #3	Inlet	1.60	23.87	0.50	0.1273	0.0008
Core 1-2 #2	Inlet	0.60	22.29	0.47	0.1254	0.0008
Site 5 VB	Outlet	5.00	0.05	0.00	0.1138	0.0020
Pond A2B	Pond A	5.00	4.63	0.03	0.1301	0.0026
Pond A2A	Pond A	5.00	5.59	0.04	0.1147	0.0019
Pond #1	Pond A	5.00	13.43	0.10	0.1043	0.0014
Canal 3a	R Canal	5.00	9.87	0.07	0.1307	0.0017
Canal 4	R Canal	5.00	12.29	0.08	0.1281	0.0020
Canal 5a	R Canal	5.00	14.63	0.11	0.1339	0.0011
Canal 5b	R Canal	5.00	12.63	0.09	0.1363	0.0016
Canal 6aB	R Canal	5.00	1.18	0.01	0.1281	0.0048
Canal 6aT	R Canal	5.00	6.32	0.04	0.1339	0.0021
